# Phylogenetic characterisation of tick-borne encephalitis virus from Lithuania

**DOI:** 10.1371/journal.pone.0296472

**Published:** 2024-02-07

**Authors:** Marina Sidorenko, Jana Radzijevskaja, Saulius Mickevičius, Maksim Bratchikov, Dalytė Mardosaitė-Busaitienė, Povilas Sakalauskas, Algimantas Paulauskas

**Affiliations:** 1 Department of Biology, Faculty of Natural Sciences, Vytautas Magnus University, Kaunas, Lithuania; 2 Department of Physiology, Biochemistry, Microbiology and Laboratory Medicine, Institute of Biomedical Sciences, Faculty of Medicine, Vilnius University, Vilnius, Lithuania; University of Bologna / Romagna Local Health Authority, ITALY

## Abstract

The Baltic states are the region in Europe where tick-borne encephalitis (TBE) is most endemic. The highest notification rate of TBE cases is reported in Lithuania, where the incidence of TBE has significantly increased since 1992. A recent study reported 0.4% prevalence of TBE virus (TBEV) in the two most common tick species distributed in Lithuania, *Ixodes ricinus* and *Dermacentor reticulatus*, with the existence of endemic foci confirmed in seven out of Lithuania’s ten counties. However, until now, no comprehensive data on molecular characterisation and phylogenetic analysis have been available for the circulating TBEV strains. The aim of this study was to analyse TBEV strains derived from *I*. *ricinus* and *D*. *reticulatus* ticks collected from Lithuania and provide a genotypic characterisation of viruses based on sequence analysis of partial E protein and NS3 genes. The 54 nucleotide sequences obtained were compared with 81 TBEV strains selected from the NCBI database. Phylogenetic analysis of the partial E and NS3 gene sequences derived from 34 Lithuanian TBEV isolates revealed that these were specific to Lithuania, and all belonged to the European subtype, with a maximum identity to the Neudoerfl reference strain (GenBank accession no. U27495) of 98.7% and 97.4%, respectively. The TBEV strains showed significant regional genetic diversity. The detected TBEV genotypes were not specific to the tick species. However, genetic differences were observed between strains from different locations, while strains from the same location showed a high similarity.

## Introduction

Tick-borne encephalitis (TBE) is an important human viral infection of the central nervous system in Europe and many parts of Asia. The etiological agent of TBE virus (TBEV) belongs to the genus *Flavivirus* of the family *Flaviviridae* [1]. Transmission of TBEV occurs only under specific environmental conditions and is dependent on a complex enzootic cycle in which small rodents of the genera *Apodemus*, *Myodes* and *Microtus* serve as reservoirs and amplifying hosts, and ixodid ticks (in Eurasia, *Ixodes ricinus* and *Ixodes persulcatus*) serve as both vectors and reservoirs [2–4].

TBEV was initially divided into three main subtypes, based on the findings of numerous studies exploring its genetic variability: European (TBEV-Eur, also known as Western) with the prototype Neudoerfl strain, Far Eastern (TBEV-FE) with the prototype Sofjin strain, and Siberian (TBEV-Sib) with the prototype Vasilchenko and Zausaev strains [5, 6]. Recently, two new subtypes Baikalian (TBEV-Bkl) [7] and Himalayan (Him-TBEV) [8] were discovered. Phylogenetic analysis has demonstrated the segregation of TBE viruses into five subtypes according to their primary geographical distribution [5]. According to phylogenetic relationships determined from the amino acid sequences of the major envelope (E) protein, the variation between TBEV-Eur, TBEV-FE and TBEV-Sib strains within subtypes is low, with a maximum of only 2.2% at the amino acid level. The maximum difference between these three subtypes has been found to be 5–6%, which is within the range of variation reported for other flaviviruses [5]. The distribution of TBEV subtypes corresponds to the ranges of their tick vectors: TBEV-Eur subtype is commonly carried by *I*. *ricinus*, while TBEV-Sib and TBEV- FE subtypes are carried by *I*. *persulcatus* ticks [6].

In recent years, the distribution area of TBEV has expanded significantly, and there has been an increasing amount of evidence showing that subtypes can be isolated outside of their nominal geographic location [9]. TBEV foci have been detected in countries or regions where the virus has not previously been observed. Novel foci have been found in Austria, Bosnia, Denmark, Moldova, Russia (the Moscow region), the Netherlands and the United Kingdom [9]. As a result, the geographical names of the TBEV subtypes do not currently reflect their strict association with a specific region, but only correspond to the greater frequency of occurrence of a particular subtype in a particular region [10]. The European TBEV subtype is common in central, northern and eastern Europe and the Baltic States. It is also found in France, South Korea, the Netherlands, and the European part of Russia [11, 12]. The Far East subtype, formerly known as the Russian spring-summer encephalitis subtype, is dominant in China, Japan and the Russian Far East. The Siberian TBEV subtype is common in Estonia, Finland, Latvia, Siberia, the Urals and other parts of Russia [13]. Different virus subtypes can circulate in the same natural area, but their prevalence varies [10]. Three subtypes of the virus (TBEV-Europe, TBEV-FE and TBEV-Sib) coexist in the Baltic countries. In addition to the three main subtypes described, new strains of TBEV have recently been identified. Deviatkin et al. [9] have recently proposed a TBEV classification based on nucleotide/amino acid data from all known TBEV sequences, indicating seven subtypes of TBEV: TBEV-Eur, TBEV-Sib, TBEV- FE, TBEV-2871 (TBEV-Ob), TBEV-Him, TBEV-178-79 (TBEV-Bkl-1) and TBEV- 886–84 (TBEV-Bkl-2). These findings clearly demonstrate that the natural diversity of TBEV is much greater than previously thought.

To date, only the European TBEV subtype has been identified in Lithuania, while three subtypes circulate together in Estonia and Latvia: TBEV-Eur, TBEV-Sib and TBEV-FE [14–20]. The *I*. *ricinus* tick, the main vector of the TBEV-Eur subtype, is widespread in Lithuania and is considered the main vector of TBEV in the country. The ranges of the two TBEV vectors, *I*. *ricinus* and *I*. *persulcatus* ticks, overlap in the eastern parts of Estonia and Latvia [21, 22]. The *I*. *persulcatus* tick, which is a vector of the TBEV-Sib and TBEV-FE subtypes [23], is not common in Lithuania as it has only been found in the north-eastern part of Lithuania near the Latvian border [24, 25]. To the best of the authors’ knowledge, this tick species has never been examined in Lithuania for the presence of TBEV. However, a study by Katargina et al. [18] has demonstrated that in areas where ranges of *I*. *ricinus* and *I*. *persulcatus* overlap, strains of TBEV-Sib and TBEV-Eur may be detected not only in natural tick vectors (*I*. *persulcatus* and *I*. *ricinus*, respectively), but in sympatric tick species as well.

A recent study reports a 0.4% prevalence of the TBEV in the two most common tick species distributed in Lithuania, *I*. *ricinus* and *D*. *reticulatus*, and confirms the existence of endemic foci in seven of Lithuania’s ten counties [26]. However, until now, no comprehensive data on molecular characterisation and phylogenetic analysis are available for the TBEV strains circulating in different parts of Lithuania. The aim of this study was to analyse TBEV strains derived from *I*. *ricinus* and *D*. *reticulatus* ticks collected from Lithuania and provide a genotypic characterisation of viruses by sequencing and phylogenetic analysis of the partial E protein and NS3 genes.

## Materials and methods

### Tick collection and detection of TBEV

Questing ticks were collected between early March and late October during 2017–2019 from 81 locations in Lithuania’s ten counties. In all, 8,846 ticks were collected and grouped into 945 pools, which were tested for the presence of TBEV. The ticks were pooled into groups by species, developmental stage, sex and location. *D*. *reticulatus* were pooled into groups of five adults (females or males), while *I*. *ricinus* were pooled into groups of 10 adults (females or males), 20 nymphs and 50 larvae. The collection of ticks and sample sites with GPS coordinates are described in more detail in Sidorenko et al. [26]. TBEV-infected *I*. *ricinus* and *D*. *reticulatus* ticks were collected from 16 sites in seven Lithuanian counties: Alytus, Kaunas, Marijampolė, Šiauliai, Telšiai, Panevėžys and Vilnius (Table 1) [26].

**Table 1:** Locations and coordinates of TBEV-positive tick samples collected in Lithuania in 2017–2019.

County	Site	year	n = x	Coordinates	Host
Alytus	Krokialaukis (Kro)	2018	4	N54°25′40′′ E23°29′5′′	*DR*, *IR*
Kaunas	Bedančiai (Bed)	2018	2	N55°27′24.4′’ E23°05′35.6′′	*IRn*
Marijampolė	Lekėčiai (Lek)	2019	1	N54°59′32.2′′ E23°27′43.0′′	*IRn*
	Želsva (Zel)	2018	2	N54°24′25,3 E23°27′28,3′′	*IR*
	Kazlų Ruda (KR)	2018	2	N54°44′13.79′′ E23°27′39.57′′	*IR*
Šiauliai	Bridai (Brd)	2018	2	N56°01′21.7 E23°19′34.8′′	*IRn*
	Suginčiai (Sug)	2018	2	N56°21′51.78′′ E22°52′26.52′′	*IR*, *IRn*
	Kairiai (Kai)	2018	1	N55°53′5,1’’ E23°25′28,4′′	*IRn*
	Kivyliai (Kyv)	2017	7	N56°21′23.84" E22°42′41.66′′	*DR*, *IR*
	Juknaičiai (Juk)	2018	1	N56°01′30.0′’ E23°42′37.6′′	*IRn*
Telšiai	Kirkliai (Kir)	2019	4	N55°57′08.3′′ E22°34′06.1′′	*IR*, *IRn*
	Mažeikiai (Maz)	2019	2	N56°22′11.12′′ E22°15′56.37′′	*IR*, *IRn*
Panevėžys	Gailiai (Gai)	2019	1	N56°17′3.78′′ E25°0′33.93′′	*IR*
	Astavas (Ast)	2019	1	N56°13′14.67′′ E24°46′3.66′′	*IR*
	Rigmantiškiai (Rig)	2019	1	N56°22′35.62′′ E24°56′14.48′′	*IR*
Vilnius	Pakalniškes (Pak)	2018	1	N54°44′13.9′′E24°41′59.9′′	*DR*

*DR*–adult *Dermacentor reticulatus*, *IR*–adult *Ixodes ricinus*, *IRn*–*Ixodes ricinus* nymph, n–number of positive pools.

RNA was extracted from the frozen ticks using the Isolate II RNA Mini Kit (Bioline, London, UK), according to the manufacturer’s instructions. Ticks were screened for the presence of TBEV RNA by amplifying the 3′ non-coding region of TBEV using quantitative Real-Time Reverse Transcription Polymerase Chain Reaction (RT-PCR), according to the protocol described by Schwaiger and Cassinotti [27] with some modifications [26].

### Amplification of partial E and NS3 genes

Tick samples that tested positive by PCR were used for one-step RT-PCR and nested PCR and for subsequent sequencing of the partial E and NS3 gene fragments. The nested PCR for the E gene was performed with outer primers 283 F1 (GAG A(T/C)C AGA GTG A(T/C)C GAG GCT GG) and 827 R1(AGG TGG TAC TTG GTT CC(A/C) TCA AGT) and inner primers 349 F2 (GTC AAG GCG KCT TGT GAG GCA A) and 814 R2 (TTC CMT CAA TGT GYG CCA CAG G) [28]. Partial TBEV NS3 gene was amplified using outer primers NS3 F1 (G(A/G)A A(T/C)G G(C/A)C T(A/G)A A(A/G)A C(T/C)A ATG A) and NS3 R1 (TGA GCT C(A/G)A C(T/C)(T/C) (T/G)CC C(A/G)T CAA) and inner primers NS3 F2 (TAY GTC AGC AGC ATT GCT CA) and NS3 R2 (TTG ATG TTT GTY CKG YTC CAT CTA T) [18].

RT-PCR amplification was carried out using the SensiFAST Probe No-ROX One-Step Kit in a total reaction volume of 25 μl reaction mixture containing 5 μl of each RNA sample, 12.5 μl 2X Reaction Mix, 1 μl RNase inhibitor, 0.5 μl Reverse Transcriptase, 0.5 μl (2.5 pmol/μl) of each outer forward and reverse primer, DNase/RNase-free water up to 20 μl. The RT-PCR and nested PCRs were performed as described in Katargina et al. [18]. The reactions were carried out at 50°C for 30 min, denaturation at 94°C for 2 min, followed by 40 cycles for 20 s at 94°C, 1 min at 60°C for the E gene and 55°C for the NS3 gene, and 1 min at 68°C, followed by an extension at 68°C for 5 min.

The nested PCR amplifications were performed in a total volume of 50 μl comprising 10 μl 5X PCR buffer, 1 μl My Taq Mix polymerase, 2.5 μl of the inner forward and reverse primers, DNase/RNase-free water and 5 μl of target DNA from the first PCR reaction. The initial denaturation was performed at 95°C for 2 min, followed by 30 cycles at 94°C for 1 min, at 65°C for 1 min for the E gene and 55°C for the NS3 gene, and at 72°C for 1 min, followed by an extension at 72°C for 10 min. Positive (TBEV-RNA of the Austrian Neudoerfl strain U27495) and negative (DNase/RNase-free water as template) controls were used in each PCR run. To minimise the potential for contamination, separate rooms were used for the first PCR and nested PCR reactions. Negative controls were included after every fifth sample in all runs. The PCR amplification products were separated using electrophoresis on a 2% agarose gel and then visualised in a UV transilluminator (UVP GelDoc-It 310 model (Ultra-Violet Products Ltd., Cambridge, UK).

PCR products were purified using the Gene Jet Gel Extraction Kit (Thermo Fisher Scientific Baltics, Vilnius, Lithuania) and sent to a sequencing service (Macrogen, the Netherlands). The Sanger sequencing technology was used to determine the sequences of partial E and NS3 genes of TBEV.

### Phylogenetic analysis

The obtained sequences were compared with selected national and international TBEV strains from the NCBI database using the BLAST and ClustalW sequence comparison algorithm. The MEGA X program [29] was used to perform sequence alignments, calculate divergence and identity between TBEV E-gene and NS3 gene sequences and construct phylogenetic trees. The evolutionary history was inferred using the maximum likelihood method. The most appropriate model of nucleotide substitution was selected based on the Bayesian information criterion values. Phylogenetic trees based on the E and NS3 genes were inferred using the Kimura 2-parameter model [30] and the Tamura-Nei model [31] with a gamma distribution of among-site variation, respectively. A phylogenetic tree based on aligned 151 amino acid sequences of E protein was constructed using the Jones-Taylor-Thornton model [32]. Bootstrap resampling with 1000 replications was used to assess the robustness of the groupings. The sequences of TBEV-Sib and TBEV-FE subtype isolates were used to confirm the European subtype classification of the isolates found in Lithuanian counties. Omsk haemorrhagic fever virus (GenBank accession no. AB507800), related to flavivirus, was chosen as the outgroup. A total of 54 TBEV sequences were submitted to the NCBI GenBank database: 25 partial E gene sequences (accession no. from MT849211 to MT849235) and 29 partial NS3 gene sequences (accession no. from MT849236 to MT849264). The gene bank accession numbers of the sequences are also given in the Supplemental material (S1 Table).

### Statistical analysis

Genetic and geographical pairwise distances, spatiotemporal analysis and mapping of TBEV genotypes were performed using the programming language R (version 4.3.2). We calculated the Pearson correlation coefficient (r) to test the linear relationship between the genetic divergence of TBEV E gene sequences and the geographic distance of tick sampling sites in Lithuania.

## Results

A total of 34 pooled tick samples positive for TBEV were analysed in this study. Twenty-nine sequences of the partial NS3 gene and 25 sequences of the partial E gene were obtained from these samples (S1 Table). Sequence analysis of the partial E and NS3 gene sequences revealed that they all belonged to the European subtype, with a maximum/minimum identity of 98.7%/98% and 97.4%/95.9% to the Neudoerfl reference strain (GenBank accession no. U27495), respectively.

In the analysis of Lithuanian TBEV strains, we also included other E and NS3 gene sequences of TBEV detected in Lithuania (GenBank accession no. AJ414703, KC660803, KC660840, KC660839, MZ964664 and MZ964665) [14, 18, 33]. Phylogenetic analysis of the E and NS3 genes included a total of 135 nucleotide sequences: the 54 sequences obtained from the present study were compared with 81 selected sequences (46 of the E gene and 35 of the NS3 gene) of TBEV strains derived from ticks, rodents and humans from the NCBI GenBank database.

### Phylogenetic analysis of the partial E gene sequences

**Fig 1:**
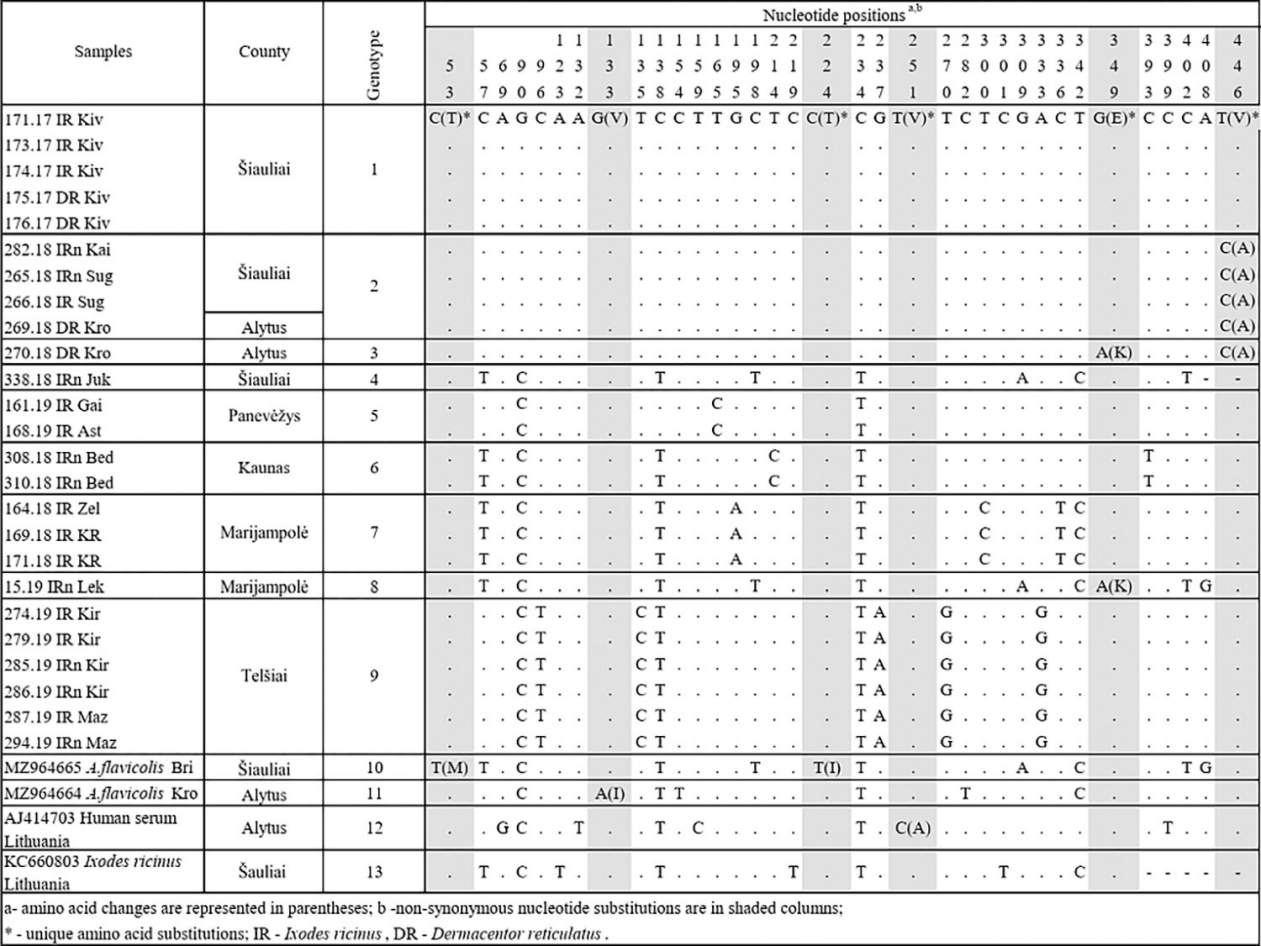
Nucleotide and amino acid substitutions in the partial E gene detected from Lithuanian TBEV strains.

A total of 29 partial E gene sequences from Lithuanian samples were analysed: 25 sequences obtained from ticks during this study (21 from *I*. *ricinus* and four from *D*. *reticulatus*) and the other four retrieved from GenBank (from *Apodemus flavicollis* mice MZ964664 and MZ964665 [33], from a human serum AJ414703 [14], and from *I*. *ricinus* tick KC660803 [18]) (Fig 1).

The analysed E gene sequences of 462 bp included thirteen variants and differed at between one to 13 nucleotide positions. Thirty-five variable nucleotides were detected, and the TBEV sequence variability was 7.57% (35 variable nucleotides/462 total nucleotides). Six of the 35 nucleotide substitutions (17.14%) were non-synonymous (Fig 1). The overall mean genetic distance between the TBEV E gene nucleotide sequences obtained in Lithuania was 0.016. The identity of the determined TBEV E gene sequences ranged from 97.1 to 100% (divergence 2.9–0.0%) at the nucleotide level (S2 Table).

**Fig 2:**
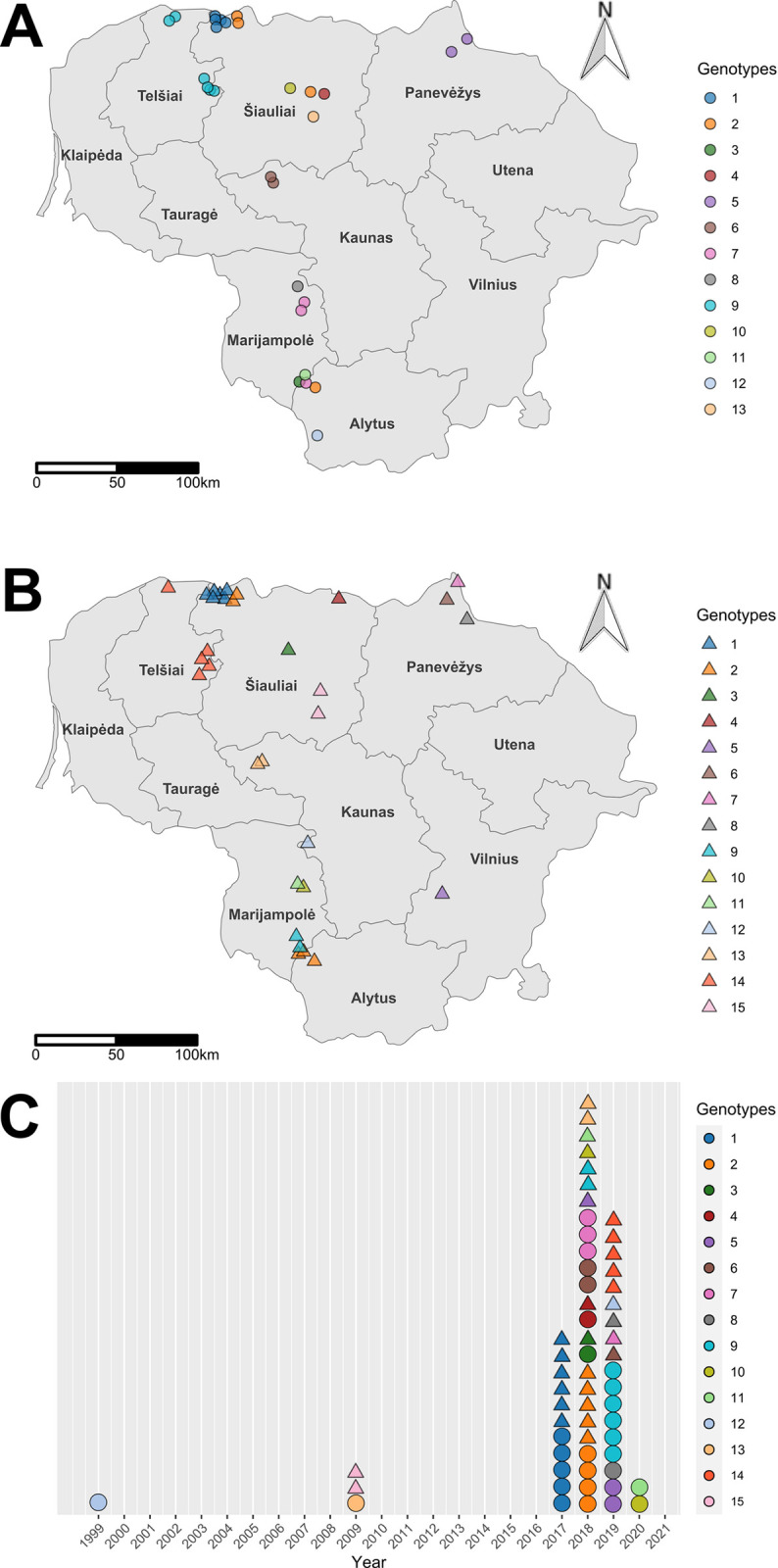
Spatial and temporal distribution of E gene and NS3 gene genotypes. In panels (A) and (B), spatial maps showcase the distribution of tested samples coloured according to their respective genotypes for the E and NS3 genes. The names of the counties are indicated on the map. To prevent overlap, samples are jittered within a range of ±15 km in both x and y directions. (C) presents the temporal distribution of E and NS3 gene genotypes, represented by circles for E gene samples and triangles for NS3 gene samples. The base map was created with Natural Earth Dataset (http://www.naturalearthdata.com/).

In order to visualise the distribution of different TBEV genotypes in Lithuania, sampling locations were plotted on the map of Lithuania and coloured according to their respective genotypes based on the partial E and NS3 gene sequence analysis. The TBEV genotypes detected based on the partial E gene and their distribution in different Lithuanian locations are presented in Figs 1 and 2.

The TBEV E gene sequences obtained from ticks collected at the same sampling site were identical in almost all cases. The exceptions were *D*. *reticulatus* ticks and *A*. *flavicolis* collected in one site in Alytus county, which harboured three different TBEV genotypes (Fig 1). The divergence between E gene nucleotide sequences obtained from *D*. *reticulatus* ticks was 0.2%, while those compared with sequence obtained from *A*. *flavicolis* (GenBank accession no. MZ964664) ranged from 1.8 to 2.0% (S2 Table). The E gene nucleotide sequences from this study and those obtained in earlier studies conducted in Lithuania [14, 18] from a human (GenBank accession no. AJ414703) and *I*. *ricinus* (GenBank accession no. KC660803) showed 97.3–98.4% and 97.2–98.6% similarity, respectively (Fig 1, S2 Table).

When the E gene sequences obtained from ticks in this study were compared with other strains circulating in Europe, the most closely related sequences were found to share 98.7–100% sequence similarity (Table 2).

**Table 2:** Comparison of the partial E and NS3 gene sequences from Lithuania with closely related sequences obtained from GeneBank.

Lithuanian counties, n = number of sequences (genotype)	Country (GenBank accession number)	Host	Percentage of identity
**E gene**			
Panevėžys, n = 2 (5)	Germany (MH704571) Estonia (MW916613) the Netherlands (MZ969639).	*D*.*reticulatus* *I*.*ricinus* *I*.*ricinus*	100%
Šiauliai, n = 8 (1, 2) Alytus n = 2 (2, 3)	Poland (KC660808)	*I*.*ricinus*	98.9 % 98.7%
Šiauliai, n = 3 (4,10, 13) Marijampolė, n = 1 (8)	Austria (KF151173)	*A*.*flavicolis*	99.3% 98.7 %
Telšiai, n = 6 (9)	Finland (MN047455)	*M*.*glareolus*	99.3%
Kaunas, n = 2 (6)	Finland (MG589937) Finland (MG589938)	*I*.*ricinus* *Human*	99.8%
Marijampolė, n = 3 (7)	Italy (MN746774) Germany (KC154191)	*I*.*ricinus* *I*.*ricinus*	99.6% 99.3%
**NS3 gene**			
Šiauliai, n = 8 (1) Alytus, n = 3 (2)	Germany (MK922615) Estonia (KC660833) the Netherlands (MZ969638)	*I*.*ricinus* *I*.*ricinus* *I*.*ricinus*	98.6–98.8%
Marijampolė, n = 4 (9,10,11)	Finland (GU183381)	*mouse*	98.3%
Telšiai, n = 5 (14)	Russia (KY069126)	*I*.* persulcatus*	99.1%
Kaunas, n = 2 (13)	Finland (MK801814) Slovakia (KC835597)	*I*.*ricinus* *M*.*glareolus*	99.1% 98.3%

**Fig 3:**
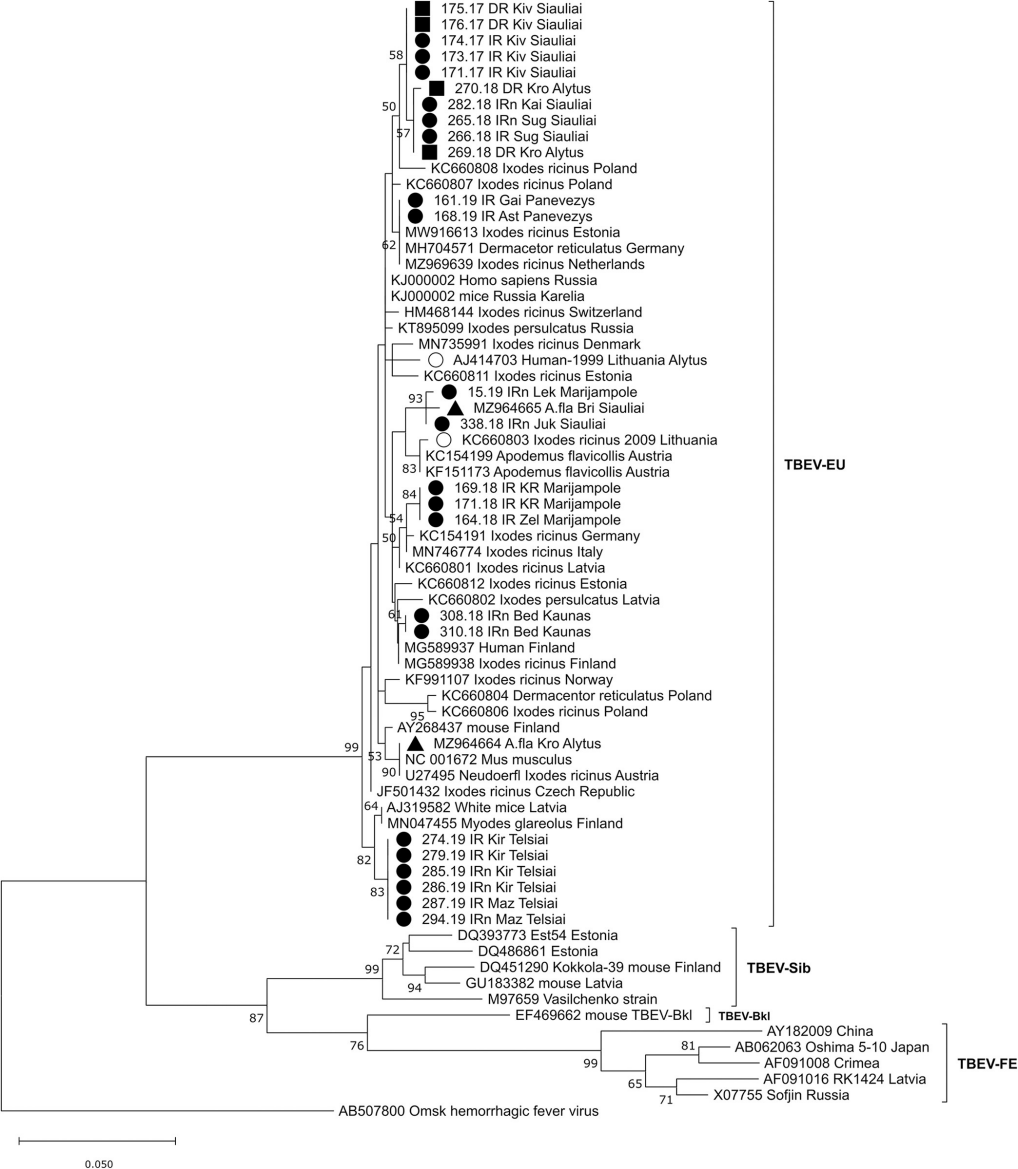
Maximum-likelihood (ML) phylogenetic tree created based on partial E gene sequences of TBEV using the Kimura 2-parameter model and bootstrap analysis of 1000 replicates (bootstrap values < 50% not shown). Sample ID or GenBank accession numbers are indicated for each sequence, with the original isolation source, location (if known) and country code. Sequences obtained from TBEV RNA-positive tick and rodent samples from six counties in Lithuania in 2017–2019 are marked as follows: obtained from ● *Ixodes ricinus*, ■ *Dermacentor reticulatus*, ▲ *Apodemus flavicollis*. *○* obtained in Lithuania from human samples in 1999 (Mickiené et al. [14]) and from ticks (Katargina et al. [18]). The scale indicates the number of nucleotide substitutions per site.

On the phylogenetic tree, Lithuanian samples split up into eight clusters (Fig 3). Most of sequences showed local geographic clustering. However, the TBEV strains from Šiauliai (northern Lithuania) and Alytus (southern Lithuania) counties clustered together, sharing 99.8–100% sequence similarity.

The E gene sequence of the TBEV strain isolated from *A*. *flavicollis* (GenBank accession no. MZ964664) in southern Lithuania in 2020 was 100% identical to the reference strain of the European subtype Neudoerfl (GenBank accession no. U27495) isolated from *I*. *ricinus* tick in Austria in 1971 (Fig 3). The similarity of the E gene nucleotide sequences from this study with Siberian subtype strains was 82.5–85.5%, and with the TBEV-FE subtype 75.9–84.2%.

**Fig 4:**
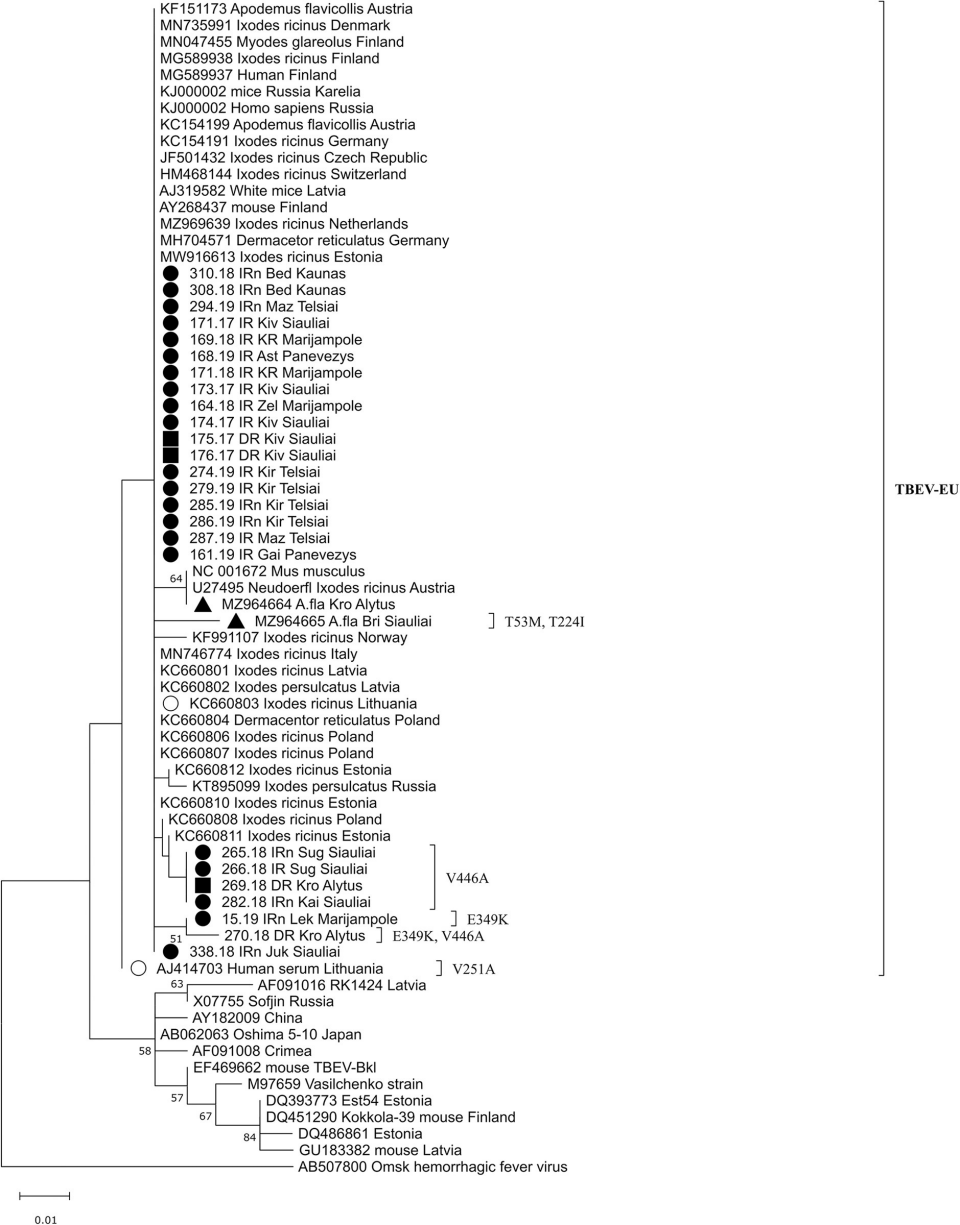
Phylogenetic tree of TBEV created based on the 151-amino-acid of E protein using ML method and Jones-Taylor-Thornton model. The scale indicates the number of amino acid substitutions per site. Amino acid substitutions are indicated by a “]” on the right side of the tree.

Amino acid sequence analysis of the Lithuanian TBEV isolates revealed the presence of six amino acid substitutions in the partial E gene: 53(T→M), 133(V→I), 224(T→I), 251(V→A), 349(E→K) and 446(V→A) (Fig 1). The five amino acid substitutions appeared to be unique among Lithuanian isolates, 53(M), 224(I), 251(A), 349(K), and 446(A), while other TBEV isolates, regardless of the subtype, had at positions 53 and 224 threonine, at 251 and 446 valine, and at 349 glutamic acid (Figs 1 and 4).

Unique amino acid substitutions were detected in eight strains derived from *I*. *ricinus* and *D*. *reticulatus* ticks, *A*. *flavicollis* mouse and a human (Figs 1 and 3).

**Fig 5:**
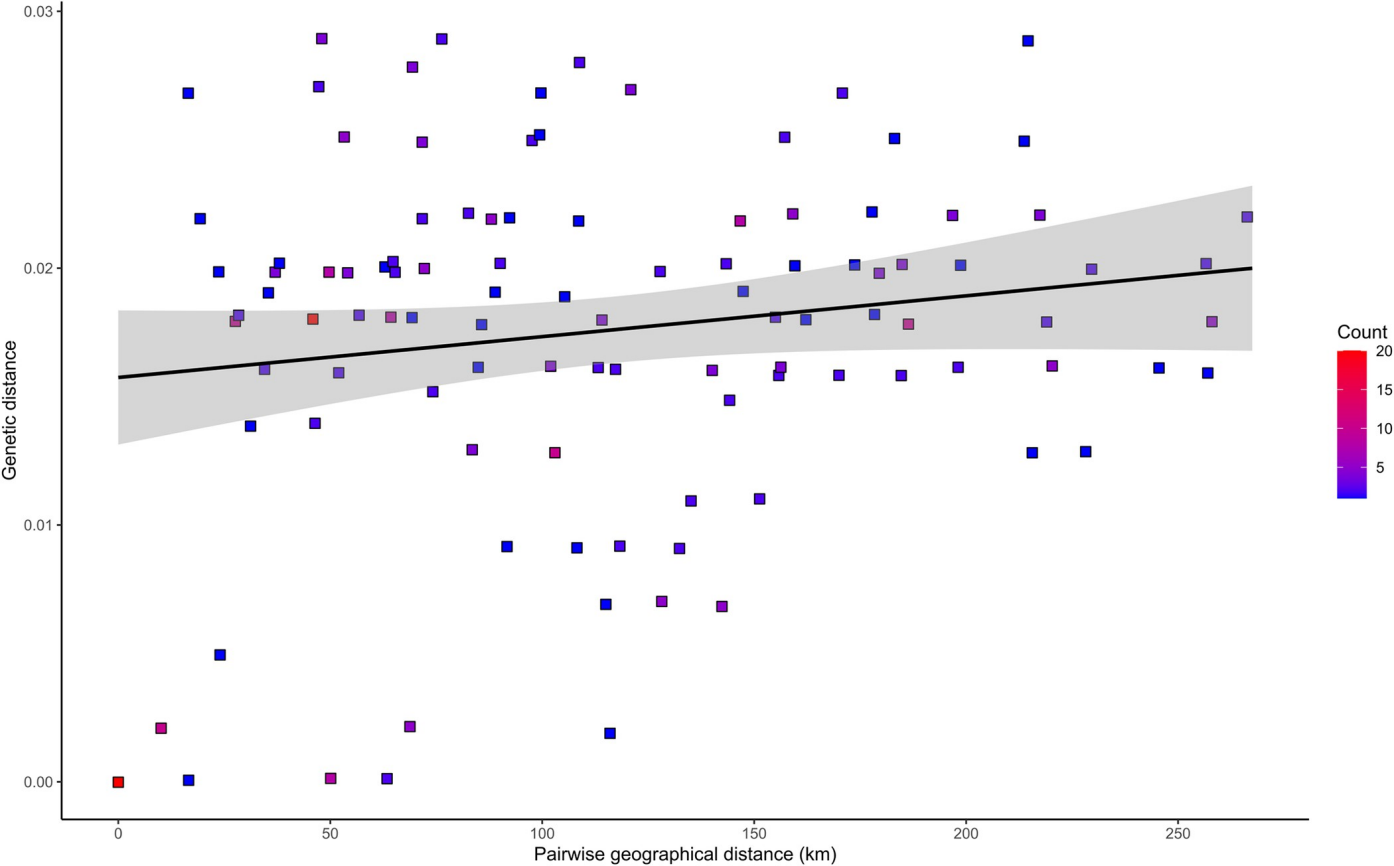
Scatter plot depicting the relationship between genetic distances of E gene sequences and corresponding geographical distances. Every dot on the plot corresponds to the pair of viruses. The y-axis shows the divergence between nucleotide sequences in the E gene fragment (462 nt) between two viruses. The distance (in km) between host collection sites for this pair is indicated on the x-axis.

To assess the potential correlation of sequence divergence to geographical locations we have checked the concordance between genetic and geographical pairwise distances. The relationships between pairwise comparisons of genetic and geographical distances for the E gene are plotted in Fig 5. Geographical pairwise distances were determined based on sampling coordinates. The maximum geographical distance between sampling sites was 257 km, and the minimum was 10 km.

The Pearson correlation revealed a low positive correlation between geographical and genetic distances (r = 0.3447) with statistical significance at p-value < 0.0001. There was evidence that the genetically diverse TBEV strains originated from distant geographical regions: genetic distance estimated among viruses from locations in southwestern Lithuanian counties Marijampolė and Alytus and viruses from the northwestern county Telšiai (distance of approximately 200 km) was 2.7–2.2% and 2.2–2.0%, respectively (Fig 2). However, genetically close viruses were isolated from ticks collected in geographically distant regions. For example, the high homology between the E gene sequences of TBEV isolates in locations of Šiauliai and Alytus counties (99.8–100% identity) was detected despite the geographic distance between these two regions of 220 km. At the same time, viruses from geographically close regions (distance of approximately 20–35 km) were genetically diverse (TBEV strains from southwestern Lithuanian counties Marijampolė and Alytus; divergence 2.7–1.6%). More than one genotype of TBEV was detected in locations in Marijampolė county (distance of approximately 28–40 km) and Šiauliai county (distance of approximately 10 km).

### Phylogenetic analysis of the partial NS3 gene sequences

**Fig 6:**
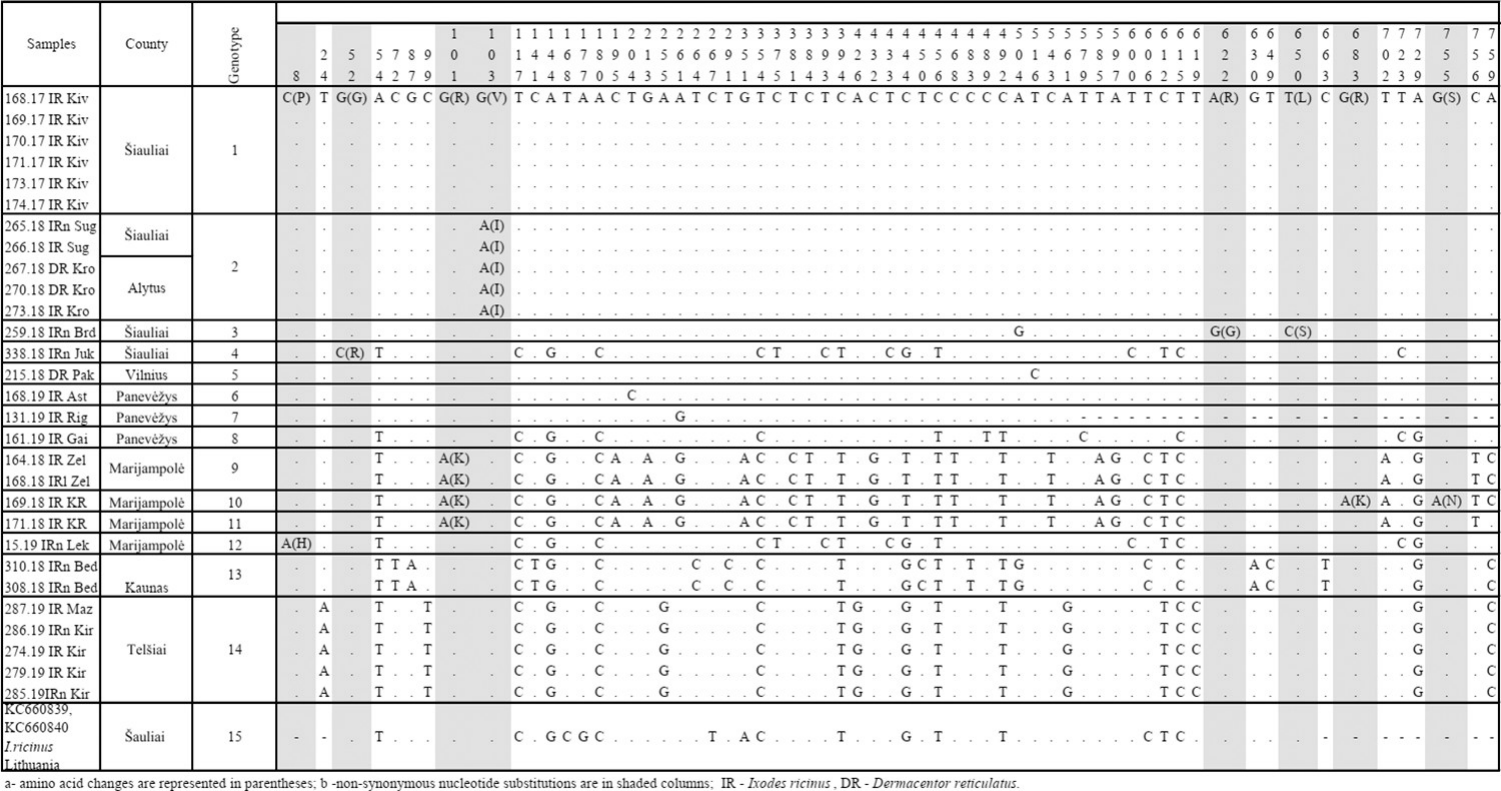
Nucleotide and amino acid substitutions in the partial NS3 gene detected among Lithuanian TBEV strains.

A total of 31 partial TBEV sequences of the NS3 gene from Lithuanian samples were analysed: 29 obtained from ticks during this study and two sequences retrieved from GenBank (from *I*. *ricinus* ticks KC660840 and KC660839 [18]). The analysed NS3 gene sequences of 761 bp have 64 variable nucleotides (sequence variability was 8.4%). Fifteen sequence variants were detected, which differed at one to 30 nucleotide positions. Eight of the 64 nucleotide substitutions (13.1%) were non-synonymous (Fig 6).

The overall mean genetic distance between the TBEV NS3 gene nucleotide sequences obtained in Lithuania was 0.021. The identity of the determined NS3 gene sequences ranged from 95.5 to 100% (divergence 4.5–0.0%) at the nucleotide level (S3 Table).

The TBEV genotypes detected based on the partial NS3 gene and their distribution in different Lithuanian locations are presented in Figs 2 and 6. All TBEV NS3 gene sequences obtained from ticks collected at the same sampling site were identical, except for one site in Marijampolė county, where two different genotypes were detected (Fig 6). The NS3 gene sequences from this study and those obtained previously in Lithuania from *I*. *ricinus* ticks [18] (GenBank accession no. KC660840 and KC660839) showed 96.9–98.4% similarity (divergence 3.1–1.6%) (S3 Table).

The NS3 protein of the Lithuanian TBEV isolates showed up to eight unique amino acid substitutions, ranging from one to three individual changes (Fig 6).

Lithuanian TBEV isolates shared 96.1–99.4% sequence similarity with the other strains of the TBEV-Eur subtype selected for comparison. The similarity of the partial NS3 gene sequences analysed in this study with sequences obtained from the GeneBank database is shown in Table 2. The similarity of the NS3 gene sequences from this study with Siberian subtype strains ranged from 82.1 to 84.5%, and with the Far East subtype ranged from 80 to 83.4%.

**Fig 7:**
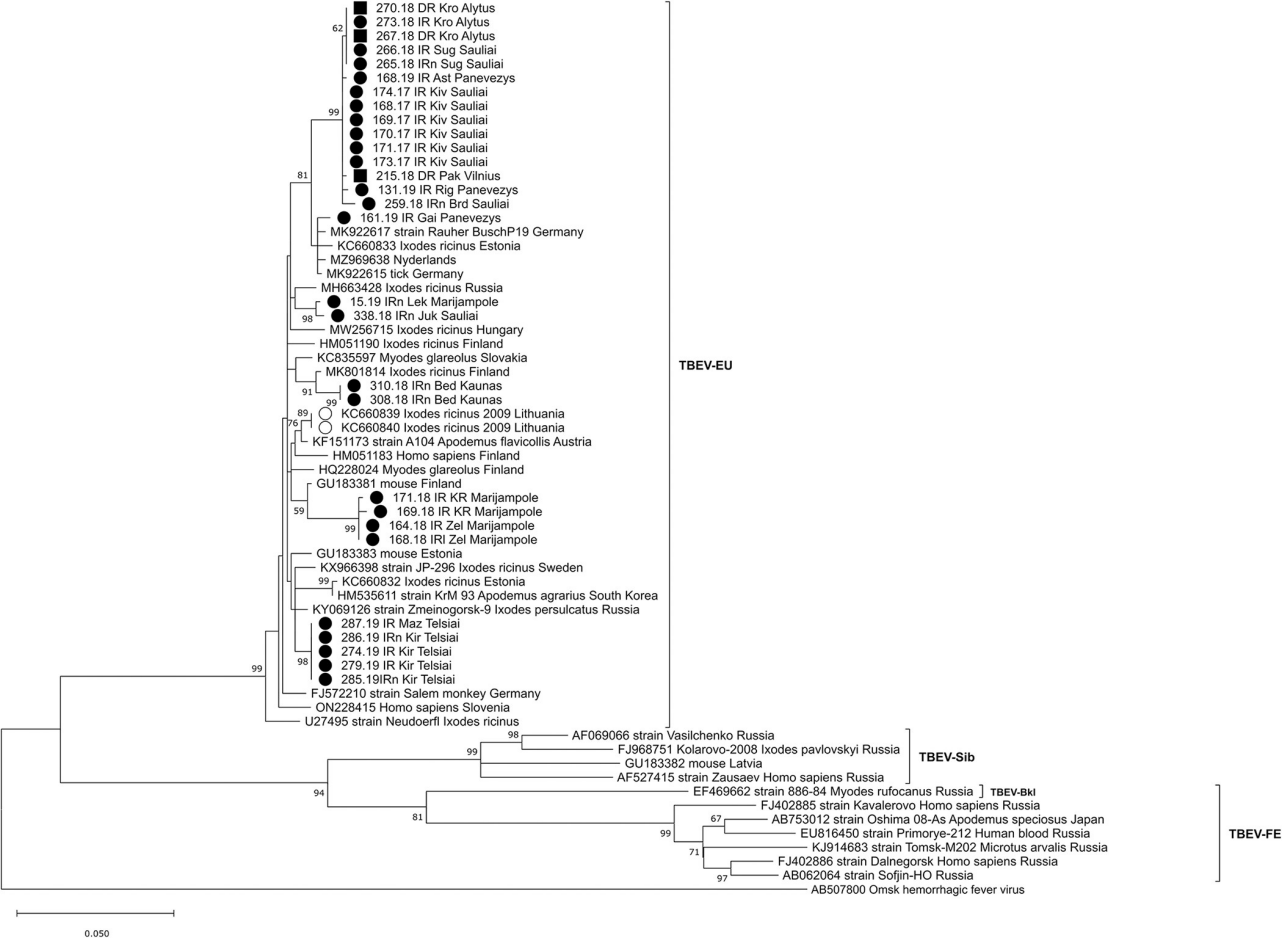
Phylogenetic tree created n based on the patrial NS3 gene sequences of TBEV using the maximum likelihood method and the Tamura-Nei model with a gamma distribution of among-site variation and bootstrap analysis of 1000 replicates (bootstrap values < 50% not shown). Sample ID or GenBank accession numbers are indicated for each sequence, with the original isolation source, location (if known) and country code. Sequences obtained from TBEV RNA–positive tick samples from seven counties in Lithuania during 2017–2019 are marked: ● –obtained from *Ixodes ricinus*; ■ –*Dermacentor reticulatus*. *○* - sequences obtained in Lithuania from ticks by Katargina et al. [18]. The scale indicates the number of nucleotide substitutions per site.

On the phylogenetic tree based on the partial NS3 gene, Lithuanian samples split up into seven clusters (Fig 7). In almost all cases, TBEV strains from the same or geographically close locations are grouped in separate clusters. Although TBEV strains from Šiauliai (northern Lithuania) and Alytus (southern Lithuania) counties originated from geographically distant regions, they shared 99.87–100% sequence similarity and, on phylogenetic tree, are grouped together (Fig 7).

## Discussion

According to the information of the Lithuanian National Public Health Centre under the Ministry of Health [34], the human risk of TBE in Lithuania is quite high, and TBE is present in all districts. The incidence of TBE has significantly increased in five out of 10 counties in Lithuania in the last two decades. According to epidemiological investigations over many years, the regions of northern (Šiauliai county) and central Lithuania (Panevėžys and Kaunas counties) are the most stable TBEV natural foci with the highest TBE incidence rates [35, 36]. A recent study indicated changes in the spatial distribution of TBE from 2005–2014 and a significant increase in disease incidence in the eastern and eastern northern parts of Lithuania [35]. Three published studies have reported the prevalence of TBEV in Lithuanian questing ticks. In an earlier study conducted by Juceviciene et al. [37], 3,234 *I*. *ricinus* and 143 *D*. *reticulatus* ticks were investigated. Ticks were collected in 2001 from different regions of Lithuania. Separate pools were made for nymphs, females and males. Each pool contained 4–10 ticks. Infected *I*. *ricinus* ticks were found in Šiauliai (0.4%), Panevėžys (0.1%) and Radviliškis (1.7%) counties. Katargina et al. [18] investigated 1,990 *I ricinus* ticks collected from four TBE-endemic regions and reported the presence of TBEV-infected ticks in Utena (0.18%) and Radviliškis (1.07%) regions. According to the recent nationwide study conducted in Lithuania in 2017–2019, which investigated 7,170 *I*. *ricinus* and 1,676 *D*. *reticulatus* ticks, TBEV-infected ticks were found at 16 locations in seven counties, with the highest TBEV infection rate detected in ticks from Alytus (1.0%), followed by Telšiai (0.9%), Šiauliai (0.8%), Marijampolė (0.7%), Panevėžys (0.3%), Kaunas (0.1%) and Vilnius (0.1%) counties [26]. The geographical spread of the virus may be explained by the emergence of new foci or the spread of existing ones [35]. The increase in TBE incidence is likely due to several factors such as changes in climate [8, 9], as well as changes in the availability of tick host species [10, 11], which all impact the tick life cycle and, thus, tick distribution [1].

It has been shown that the microclimate and the coincidence of tick and host population densities are the two main factors influencing the focal occurrence of TBEV in ticks [38]. On the other hand, human-induced factors, such as agro-economical changes in the use of land, intensive farming, changes in infrastructure and other aspects of anthropogenic activity are also likely to affect the populations of ticks and their hosts. A previous comprehensive study conducted in the Baltic States investigated the biological and non-biological causes of the spatial heterogeneity and temporal change of TBE within Estonia, Latvia, and Lithuania in 1993–1998 (immediately after TBE incidence had increased to its highest level). It was found that 55% of the observed spatial variation in TBE incidence across all Baltic States could be explained by the land cover and seasonal patterns of climatic indices. A specific change in spring temperature conditions during this period may have enhanced TBEV transmission [39].

Although TBE is a serious problem in Lithuania, detailed information concerning local virus strains and their genetic variability remains limited. Only a few studies have been conducted in Lithuania in which partial genome sequences of E, NS3 and NS5 genes and the NCR region of TBEV have been analysed [14, 15, 17, 18, 20]. The first isolation and partial genetic characterisation of a TBEV strain from a Lithuanian patient’s serum sample was performed in 1999 [14]. The patient had been bitten by a tick in the Lazdijai district in the southernmost part of Lithuania (Alytus county). The partial E gene sequence (GenBank accession no. AJ414703) recovered from the TBEV isolate showed the closest similarity to the TBEV-Eur subtype and shared 97.1–98.4% similarity with isolates derived in 2017–2020 from ticks and rodents (and was 97.8–98% identical with TBEV strains identified in Alytus county in 2018 and 2020) (S2 Table). The first genetic characterisation of TBEV isolates from *I*. *ricinus* ticks was performed in 2005 by Han et al. [17]. Phylogenetic analysis was based on a small number of analysed Lithuanian sequences: five partial NS5 sequences (GenBank accession no. DQ112086, DQ112087, DQ112088, DQ112089 and DQ112090) and one partial E gene sequence (GenBank accession no. DQ112085) from the regions of Šiauliai and Radviliškis. Similarly, as detected in the present study, phylogenetic analysis showed that TBEV NS5 sequences were identical within one locality (Radviliškis region). The genetic variation between strains from different locations was small (differing by one nucleotide) [17]. Phylogenetic analysis based on the nucleotide sequences of the TBEV E and NS3 genes derived from *I*. *ricinus*, *I*. *persulcatus* and *D*. *reticulatus* ticks collected in 2006–2009 from the three Baltic states and Poland was performed by Katargina et al. [18]. In this study, only one E gene (GenBank accession no. KC660803) sequence and two identical NS3 gene (GenBank Accession no. KC660839 and KC660840) sequences belonging to the TBEV-Eur subtype derived from two *I*. *ricinus* specimens from Lithuania were analysed. On the phylogenetic trees, sequences of the TBEV-Eur subtype obtained from ticks in Latvia, Lithuania, and Poland did not show geographical clustering within the TBEV-Eur subtype [18].

The present study compared nucleotide sequences of the E and NS3 genes of 39 TBEV strains isolated from Lithuania over a period of 20 years (34 strains isolated from ticks in 2017–2019, two strains from rodents in 2019, one strain from humans in 1999 and two strains from ticks in 2009). The samples collected in different years originated from different locations (Table 1). The phylogenetic analysis indicated that all strains for the TBEV E gene and NS3 gene sequenced in this study were dispersed among the other isolates of the TBEV-Eur subtype from different countries and were clearly different from the strains of the TBEV-Sib and TBEV-FE subtypes (Figs 3 and 7). The phylogenetic trees based on the partial E and NS3 genes generally represented the same branch pattern and similarity among the Lithuanian strains analysed (Figs 3 and 7). The TBEV isolates from ticks and rodents collected in 2017–2020 showed significant regional genetic diversity: 11 variants with 27 variable nucleotides were detected among the 27 E gene sequences, and 14 variants with 61 variable nucleotides were detected among the 29 NS3 gene sequences. The TBEV strains from Lithuania had unique amino acid substitutions in the E and NS3 gene regions (Figs 1 and 6). The identity of the determined TBEV E and NS3 gene sequences ranged from 97.1 to 100% and 95.5 to 100% at the nucleotide level, translating into a homology of 98–100% and 98.4–100% at the amino acid level, respectively.

Most of the detected TBEV strains were specific to Lithuania. However, we found 100% of identity in partial E gene sequence among strains from Baltic countries (Estonia (MW916613) and northeastern Lithuanian region (161.19 IR Gai, 168.19 IR Ast; Panevežys county)) and TBEV strains from Germany and the Netherlands (long considered a nonendemic country for TBEV) (Fig 3). Viruses with very similar sequences collected in regions separated by more than a thousand kilometers were likely recently introduced into novel territories. These findings confirm the recent westward spread of TBEV in Europe. A recent study conducted in the Netherlands indicated that local TBEV strains were more closely related to strains from England, Germany and Sweden than to each other [4].

The phylogenetic analysis of the partial E gene of Lithuanian TBEV strains showed diverse patterns in phylogenetic grouping. When the relationships between pairwise comparisons of genetic and geographical distances for the E gene were assessed, a low statistically significant correlation between geographical and genetic distances was detected. However, we found that some viruses obtained from ticks collected from geographically close locations (Alytus and Marijampolė counties) were genetically more diverse than viruses obtained from ticks collected in geographically distant regions (Alytus and Šiauliai counties).

Several possibilities that influence the spread of TBEV in Lithuania could be discussed. Continuous spread (for a short distance), which is associated with terrestrial transport of TBEV-infected ticks by wild animal hosts such as roe deer or wild boar, and discontinuous spread (for a long-distance transfer) which is associated with aerial transport of TBEV-infected ticks by migratory birds [38, 40–42]. Landscape features and anthropogenic factors such as agricultural intensification, deforestation, and urbanization could also influence TBEV spread.

Agro-economical changes in the use of land, intensive farming, and other aspects of anthropogenic activity which cause habitat fragmentation may affect ticks and their host populations and the continuous spread of TBEV in Lithuania. Road networks and traffic fragment habitats create barriers and prevent dispersal. A recent study investigated the impact of roadkill on overall cervid populations (including moose, red deer and roe deer) in Lithuania. The study showed that the number of roe deer (*Capreolus capreolus*) killed on the roads from 2014–2020 increased exponentially [43]. The European “Via Baltica” highway is one of the most intensive transit traffic roads in Lithuania, which connects Lithuania and neighbouring Poland. This motorway stretches from the south to the north of Lithuania (goes through Panevežys, Kaunas and Marijampolė counties). It was demonstrated that Via Baltica could have an impact on the population structure of raccoon dogs and wild boars in Lithuania [44, 45]. This motorway could be a barrier to the continuous spread of TBEV-infected ticks between neighbouring regions of Alytus and Marijampole counties. In addition, Marijampolė county (where different genotypes of TBEV were detected) is one of the most intensive areas of agricultural production in Lithuania. Large-scale, intensive, monoculture agriculture leads to an overall simplification of the environment and reduction in total biodiversity, alters the vegetation and microclimate that is essential for tick survival and causes declines in wildlife species that may serve as tick hosts through the direct loss of habitat [46].

In Lithuania, the ungulate game animals, including the two most widespread species in the country, roe deer (*C*. *capreolus*) and wild boar (*Sus scrofa*), are important hosts for *Ixodes ricinus* and could be involved in the continuous spread of TBEV. Several studies demonstrate the potential contribution of wild boar to high incidence and the local and regional spreading of TBE [47–49]. Wild boar was suggested to be suitable sentinels to estimate TBEV seroprevalence in endemic areas. According to data from Belgium, Germany, and the Netherlands, up to 33% of wild boars have TBEV-positive serum samples [48–50]. In Lithuania, the abundance of wild boar did not differ significantly until 2014 (according to the monitoring data, in 2014, the wild boar population exceeded 60,000 individuals). However, the emergence of African swine fever (ASF) in Lithuania and its subsequent persistence has led to a decline in the wild boar population to 22,000 individuals [45]. The genetic structure of the wild boar population was affected by ASF: populations in the western part of Lithuania with no ASF infections were found to be genetically distinct from populations in other parts of the country affected by ASF [45]. Land cover types, such as forest and water (maritime wetlands, inland wetlands, coastal lagoons and estuaries), were found to affect the occurrence of ASF in wild boar in the Baltic States [51]. A recent study conducted in Belgium found the highest TBEV prevalence (33%) in the area with the largest mixed deciduous woods (with a predominance of red American oak) in the country. This area provides an ideal habitat for *I*. *ricinus* ticks and appropriate conditions to sustain a rising wild boar population [50].

The high homology of some TBEV strains from northern Lithuania and southern Lithuania in their E and NS3 gene sequences suggests discontinuous distribution patterns of TBEV strains via infected tick migration by avian hosts (migratory birds) [42]. Birds play an important role in the long-range and short-range migration of *I*. *ricinus* in Europe [52]. The Baltic Flyway, stretching from Scandinavia and Northern Russia down through the Baltic states, is among the most important bird migration routes in the whole world. There are two dominant birds’ migratory routes in Lithuania [53, 54]. Bird migration occurs during spring, summer and autumn periods on a broad front covering the whole of Lithuania. Up to 70% of the birds of the southwestern migration pass through the territory of Lithuania in autumn. Data from Lithuania, where a total of 1262 ticks from 22 bird species were collected from 5099 birds in 2016–2018 years at Ventės Ragas ornithological station, showed that the main carriers of ticks were blackbirds, redwings, song thrushes, dunnocks and European robins with *I*. *ricinus* identified as predominant species [55].

This study is the first to describe TBEV strains from *D*. *reticulatus* ticks in Lithuania. In an earlier study conducted in Lithuania [26], the TBEV was found with a similar prevalence in sympatric populations of *D*. *reticulatus* and *I*. *ricinus* ticks. TBEV-Eur strains isolated from *D*. *reticulatus* in this study were not specific to this tick species and had a high degree of sequence homology with the strains isolated from *I*. *ricinus* ticks. On the phylogenetic trees based on partial E and NS3 genes, TBEV strains from *D*. *reticulatus* did not form their own lineages but instead clustered with the strains isolated from *I*. *ricinus* ticks. A similar finding was detected in a study conducted in Germany [56]. It was suggested that such similarity of strains from different tick species might indicate a recent introduction of TBEV strains into the population of *D*. *reticulatus*, and the TBEV strains have not yet adapted to different tick species belonging to different tick genera. During the past two decades *D*. *reticulatus* has expanded its range in the Baltic countries during the past two decades. TBEV-positive *D*. *reticulatus* ticks analysed in this study were collected in locations where this species was not found thirty years ago [25].

## Conclusions

In Lithuania, TBEV seems to have a focal distributional pattern in endemic areas, as the virus is not uniformly present in the tick population [18, 26, 37]. Genetic analysis revealed the presence of specific TBEV strains in certain regions. TBEV strains from the same sampling site were identical in almost all cases. However, genetic differences in the E and NS3 gene sequences were observed between TBEV strains originating from different geographical regions of Lithuania, except for some strains from northern and southern regions, which were identical. None of the newly detected strains from 16 sampling sites were grouped with the TBEV strain previously detected from Lithuania, partly because of a lack of studies using NS3 and E genes of viral genomes in Lithuania. The present study indicates that at least six distinct virus lineages are circulating in ticks and two in rodents in Lithuania. Thus, further studies on whole-genome sequencing are required to gain a better understanding of the regional genetic diversity of TBEV strains.

## Supporting information

S1 Table GenBank nucleotide accession numbers of the E gene and NS3 gene sequences of the TBEV strains derived from ticks in Lithuania. (DOCX)

S2 Table Estimates of evolutionary divergence between partial E gene sequences from Lithuanian samples of TBEV. (XLS)

S3 Table Estimates of evolutionary divergence between partial NS3 gene sequences from Lithuanian samples of TBEV. (XLS)
