# Supplementary material for: Phylogenetic characterisation of tick-borne encephalitis virus from Lithuania
Source: PLoS One. 2024 Feb 7;19(2):e0296472. doi: 10.1371/journal.pone.0296472 (PMC10849421; doi:10.1371/journal.pone.0296472)
Supplement: S1 Table — (DOCX) [file pone.0296472.s001.docx]

**S1 Table.** GenBank nucleotide accession numbers of the E gene and NS3 gene sequences of the TBEV strains derived from ticks in Lithuania.

| E gene | | | | NS3 gene | | | |
| --- | --- | --- | --- | --- | --- | --- | --- |
| No. | **Strain designation** | **Accession no.** | **Isolation source** | **No.** | **Strain designation** | **Accession no.** | **Isolation source** |
| 1 | 274.19_IR_Kir | MT849211 | *IR* | **1** | 338.18_IRn_Juk | MT849236 | *IRn* |
| 2 | 269.18_DR_Kro | MT849212 | *DR* | **2** | 168.17_IR_Kiv | MT849237 | *IR* |
| 3 | 282.18_IRn_Kai | MT849213 | *IRn* | **3** | 169.17_IR_Kiv | MT849238 | *IR* |
| 4 | 164.18_IR_Zel | MT849214 | *IR* | **4** | 170.17_IR_Kiv | MT849239 | *IR* |
| 5 | 169.18_IR_KR | MT849215 | *IR* | **5** | 171.17_IR_Kiv | MT849240 | *IR* |
| 6 | 171.18_IR_KR | MT849216 | *IR* | **6** | 173.17_IR_Kiv | MT849241 | *IR* |
| 7 | 265.18_IRn_Sug | MT849217 | *IRn* | **7** | 174.17_IR_Kiv | MT849242 | *IR* |
| 8 | 266.18_IR_Sug | MT849218 | *IR* | **8** | 164.18_IR_Zel | MT849243 | *IR* |
| 9 | 270.18_DR_Kro | MT849219 | *DR* | **9** | 168.18_IR_Zel | MT849244 | *IR* |
| 10 | 171.17_IR_Kiv | MT849220 | *IR* | **10** | 169.18_IR_KR | MT849245 | *IR* |
| 11 | 173.17_IR_Kiv | MT849221 | *IR* | **11** | 171.18_IR_KR | MT849246 | *IR* |
| 12 | 174.17_IR_Kiv | MT849222 | *IR* | **12** | 215.18_DR_Pak | MT849247 | *DR* |
| 13 | 175.17_DR_Kiv | MT849223 | *DR* | **13** | 259.18_IRn_Brd | MT849248 | *IRn* |
| 14 | 176.17_DR_Kiv | MT849224 | *DR* | **14** | 265.18_IRn_Sug | MT849249 | *IRn* |
| 15 | 161.19_IR_Gai | MT849225 | *IR* | **15** | 266.18_IR_Sug | MT849250 | *IR* |
| 16 | 168.19_IR_Ast | MT849226 | *IR* | **16** | 267.18_DR_Kro | MT849251 | *DR* |
| 17 | 279.19_IR_Kir | MT849227 | *IR* | **17** | 270.18_DR_Kro | MT849252 | *DR* |
| 18 | 285.19_IRn_Kir | MT849228 | *IRn* | **18** | 273.18_IR_Kro | MT849253 | *IR* |
| 19 | 286.19_IRn_Kir | MT849229 | *IRn* | **19** | 310.18_IRn_Bed | MT849254 | *IRn* |
| 20 | 287.19_IR_Maz | MT849230 | *IR* | **20** | 308.18_IRn_Bed | MT849255 | *IRn* |
| 21 | 294.19_IRn_Maz | MT849231 | *IRn* | **21** | 287.19_IR_Maz | MT849256 | *IR* |
| 22 | 308.18_IRn_Bed | MT849232 | *IRn* | **22** | 286.19_IRn_Kir | MT849257 | *IRn* |
| 23 | 310.18_IRn_Bed | MT849233 | *IRn* | **23** | 274.19_IR_Kir | MT849258 | *IR* |
| 24 | 338.18_IRn_Juk | MT849234 | *IRn* | **24** | 279.19_IR_Kir | MT849259 | *IR* |
| 25 | 15.19_IRn_Lek | MT849235 | *IRn* | **25** | 285.19_IRn_Kir | MT849260 | *IRn* |
|  |  |  |  | **26** | 168.19_IR_Ast | MT849261 | *IR* |
|  |  |  |  | **27** | 161.19_IR_Gai | MT849262 | *IR* |
|  |  |  |  | **28** | 15.19_IRn_Lek | MT849263 | *IRn* |
|  |  |  |  | **29** | 131.19_IR_Rig | MT849264 | *IR* |

*DR –* adult *Dermacentor reticulatus, IR –* adult *Ixodes ricinus, IRn* – *Ixodes ricinus* nymph
